# Measurement inaccuracies in X-ray planning of deformity correction using a new hexapod calibration device: an experimental approach with CT-correlation

**DOI:** 10.1007/s00402-025-06105-2

**Published:** 2025-10-29

**Authors:** Moritz Mederake, Marc-Daniel Ahrend, Gabriel Keller, Heiko Baumgartner

**Affiliations:** 1 Department of Traumatology and Reconstructive Surgery, BG Trauma Center Tübingen, Eberhard-Karls University of Tübingen, Schnarrenberg-Str. 95, 72076 Tübingen Germany; 2 Universitätsklinikum Tübingen, Tübingen, Germany

**Keywords:** Bone lengthening, Deformity correction, Hexapod, Planning software

## Abstract

**Introduction:**

Hexapod systems are accurate devices to perform deformity correction. Crucial to achieving the desirable result is the precise determination of the mounting and deformity parameters. Smith + Nephew (London, UK) developed a calibration device for their hexapod system TSF (Taylor Spatial Frame) called Beacon, which is directly mounted to the reference ring. The software indicates the Beacon in X-rays and calculates the whole hexapod construct. However, we still can find inaccuracies after completing the correction plan by the software. Since the software calibrates the construct with the bone in the center of the ring, the question arises as to whether different positions of the bone within the construct influence the calculated parameters.

**Materials and methods:**

A tibial artificial bone (Sawbones, Vashon Island, USA) was mounted with the TSF. Iron beads were attached to define reference distances. The test setup included five different positions of the bone. X-rays and CTs (computed tomography) were performed and the distances were measured. The accuracy of the measurements of the X-rays and the CT were compared when having the bone in different positions.

**Results:**

Measurements were dependent on the position of the bone in relation to the center of the ring. If the bone is closer to the detector than the center of the ring, the measured distance is shorter and vice versa. Resultingly, the measured total length of the bone in X-rays varied from the true length between ± 26 mm (7%). In contrast, measurements by CT showed much less variability.

**Conclusion:**

When using X-ray planning, care must be taken if the bone is not centered in the reference ring. Measurement inaccuracies of up to 7–10% may occur. CTs give more accurate measurements regardless of the bone position. These findings should be taken into account if the limb cannot be centered in the reference ring of the hexapod system.

## Introduction

A hexapod system like the TSF (Taylor Spatial Frame, Smith + Nephew, London, UK) is one of the most accurate devices to perform bone lengthening procedures or deformity correction [1]. Its accurate treatment results as well as the proven accuracy in the treatment of non-unions or complex deformities are known [2]. Crucial to achieving the desirable result is the correct use of the correction planning software and the precise determination of the mounting and deformity parameters. Furthermore, the exact determination of the center of rotation and angulation (CORA) in relation to the reference ring is mandatory. Incorrect measurements of these parameters can lead to inaccurate deformity correction, which in turn leads to persistent or new deformities [2–6]. Ferreira et al. showed that using the latest version of the SMART-TSF Web Application (Smith + Nephew, London, UK) with the corresponding latest device, the Beacon (Smith + Nephew, London, UK), manual radiographic analysis can be accurate and represents the gold standard for deformity analysis [7]. Moreover, the SMART-TSF X-ray analysis is considered to be quick and accurate for clinical practice. However, in our daily clinical routine we experienced small discrepancies between the measured parameters with the calculated corrections and the actual necessary corrections for a good clinical outcome.

A previously conducted radiological study was able to show a high variability of the measured leg length depending on the position of the magnification device in relation to the detector [1, 2]. 76% of patients had a measured limb length difference of ≥ 2 cm depending on the sagittal positioning of the magnification device in relation to the bone. Furthermore, the position of the leg in relation to the detector is important, since different rotations can produce varying results [1]. Therefore, a standardized distance between detector and magnification device as well as a standardized position of the leg in relation to the detector are mandatory for reliable results. The problem regarding a standardized position of the magnification device seems to be solved with the recently introduced Beacon by attaching the device to the TSF reference ring and the three-dimensional (3D) digital detection of it. The position is registered in the SMART-TSF Web Application and the reference ring as well as the whole hexapod construct can be shown in 3D. It also can be projected over the X-rays in the software to perform the digital deformity correction. However, even using a standardized position with the Beacon on the ring, the distance between the Beacon and the leg can vary, since the leg is not always in the center of the ring. Having a closer look on the possibilities of mounting, the bone can be mounted in different positions in relation to the ring. This might result in inaccuracies during the correction process.

To minimize the influence of the position of the magnification device in relation to the bone in plain radiographs, the simplest option is to always mount the bone in the center of the ring, which is practically impossible due to the individual anatomy. Alternatively, CTs can be used to plan deformity correction. Having a 3D-imaging method there is no need of a magnification device, and the described distances and positions can be directly measured in the CT images.

Therefore, the aim of the study was to assess the accuracy of radiographic deformity correction planning using the Beacon with its´ associated SMART-TSF Web Application. Particular attention was paid to the position of the bone in relation to the center of the ring. The accuracy of X-ray planning was compared to CT-based planning.

## Materials and methods

### Test specimen

**Fig. 1:**
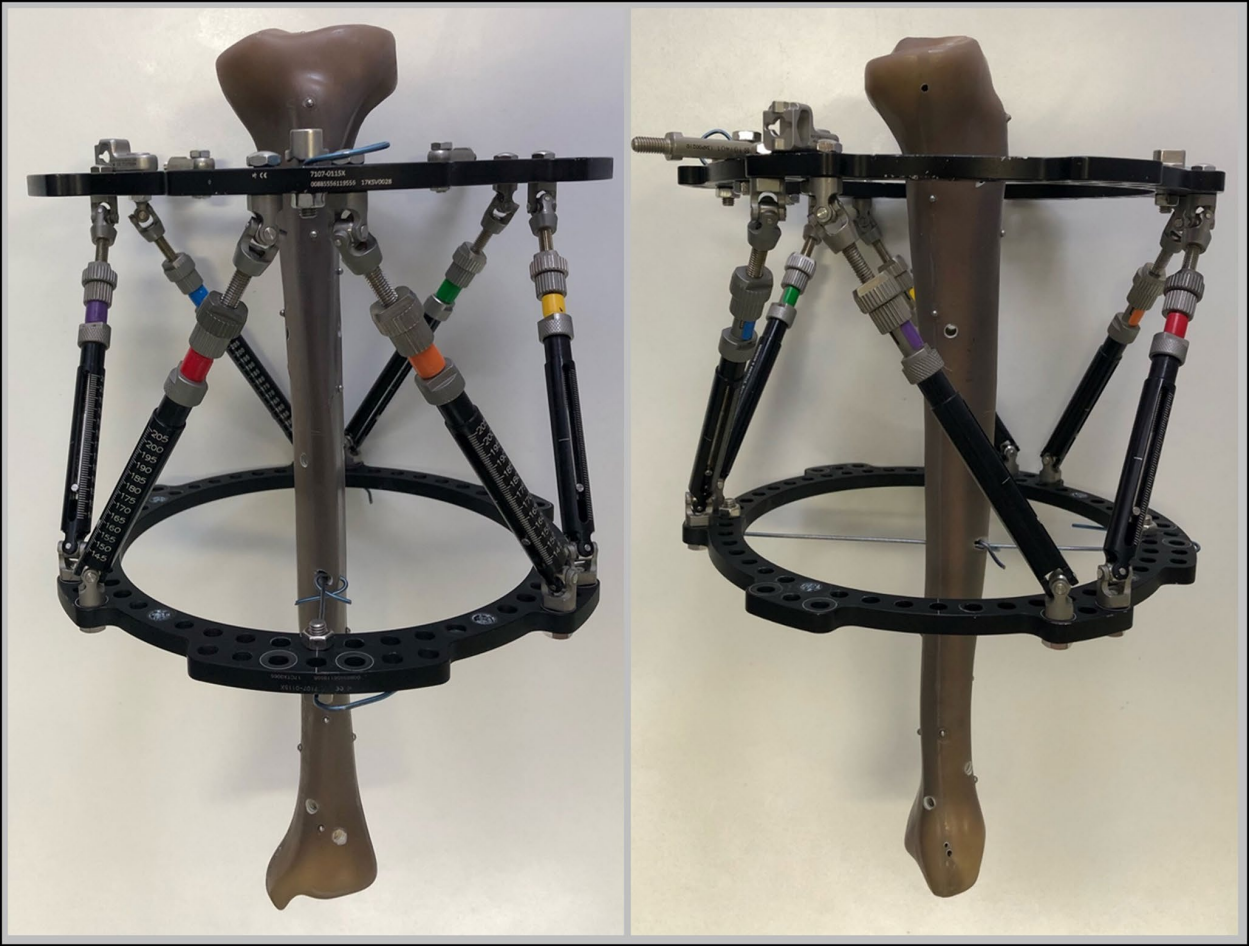
Experimental setup with the tibial bone (Tibia, Short Fiber Filled 4th Generation, Composite Bone, 102 PCF Solid Foam Core, Sawbones, Vashon Island, USA) mounted with the Taylor Spatial Frame (Smith + Nephew, London, UK)

A left tibial artificial bone (Tibia, Short Fiber Filled 4th Generation, Composite Bone, 102 PCF Solid Foam Core, Sawbones, Vashon Island, USA) was mounted within the TSF. Two rings with 180 mm were fixed to the bone with two K-wires and wire tension clamps each. Measured by a sliding caliper, the center of the proximal ring was mounted 54 mm distal to the tibial plateau and the center of the distal ring was 158 mm proximal to the joint line of the ankle. Both rings were mounted perpendicular to the bone in the coronal and the sagittal plane and held in position with six struts. Settings of all the struts were 155 mm (Fig. 1).

**Fig. 2:**
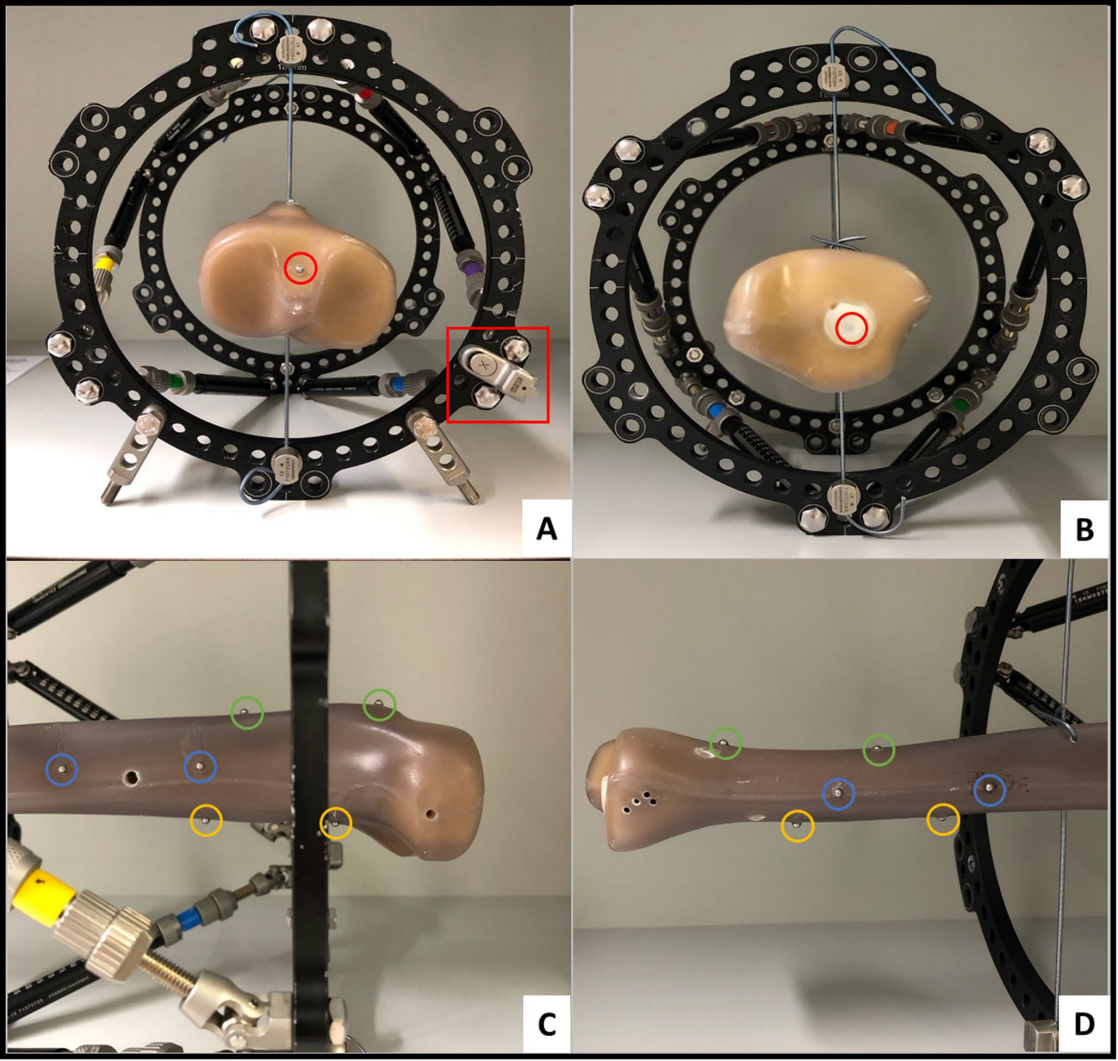
Tibial bone with reference distances marked with iron beads. **A** Iron bead (red circle) to mark the proximal bone end. Standard Beacon position between strut 5 and 6 (red square). **B** Iron bead (red circle) to mark the distal bone end. **C** 50 mm reference distances on the level of the proximal ring marked with iron beads ventral (green circle), lateral (blue circle) and dorsal (orange circle). The medial reference distance is not visible. **D** 50 mm reference distances on the level of the distal ring marked with iron beads ventral (green circle), lateral (blue circle) and dorsal (orange circle). The medial reference distance is not visible

To measure distances on X-rays, iron beads with a diameter of 2 mm were attached to the bone in defined positions (reference distances). One bead each was positioned on the proximal and distal joint line to be able to measure the total bone length. Furthermore, on the level of the proximal and the distal ring, respectively, eight 50 mm reference distances (ventral, dorsal, medial and lateral on the bone) were marked with two beads each. The standard Beacon position was dorsomedial (between strut 5 and 6) (Fig. 2).

### Test setup

To be able to analyze the influence of the position of the bone within the ring system, the test setup included five different positions of the bone with the Beacon in standard position (between strut 5 and 6). There was no change in strut length. The bone was fixed in the center of the ring with a K-wire proximal and distal for fixation. K-wires were fixed at opposite positions on the ring and aimed through the center of the bone for centering.

At first, the K-wires run from medial to lateral and were tensioned with 100 Nm. The anteroposterior centering was confirmed with the sliding caliper. After obtaining the X-rays and the CT, the bone was moved to the medial, then to the lateral position without loosening the K-wires. After imaging in these two positions, the bone was again centered in the ring. Then two new K-wires were applied through the center anterior and posterior direction. The first K-wires were removed and the bone could be moved to the ventral and then to the dorsal position.

**Fig. 3:**
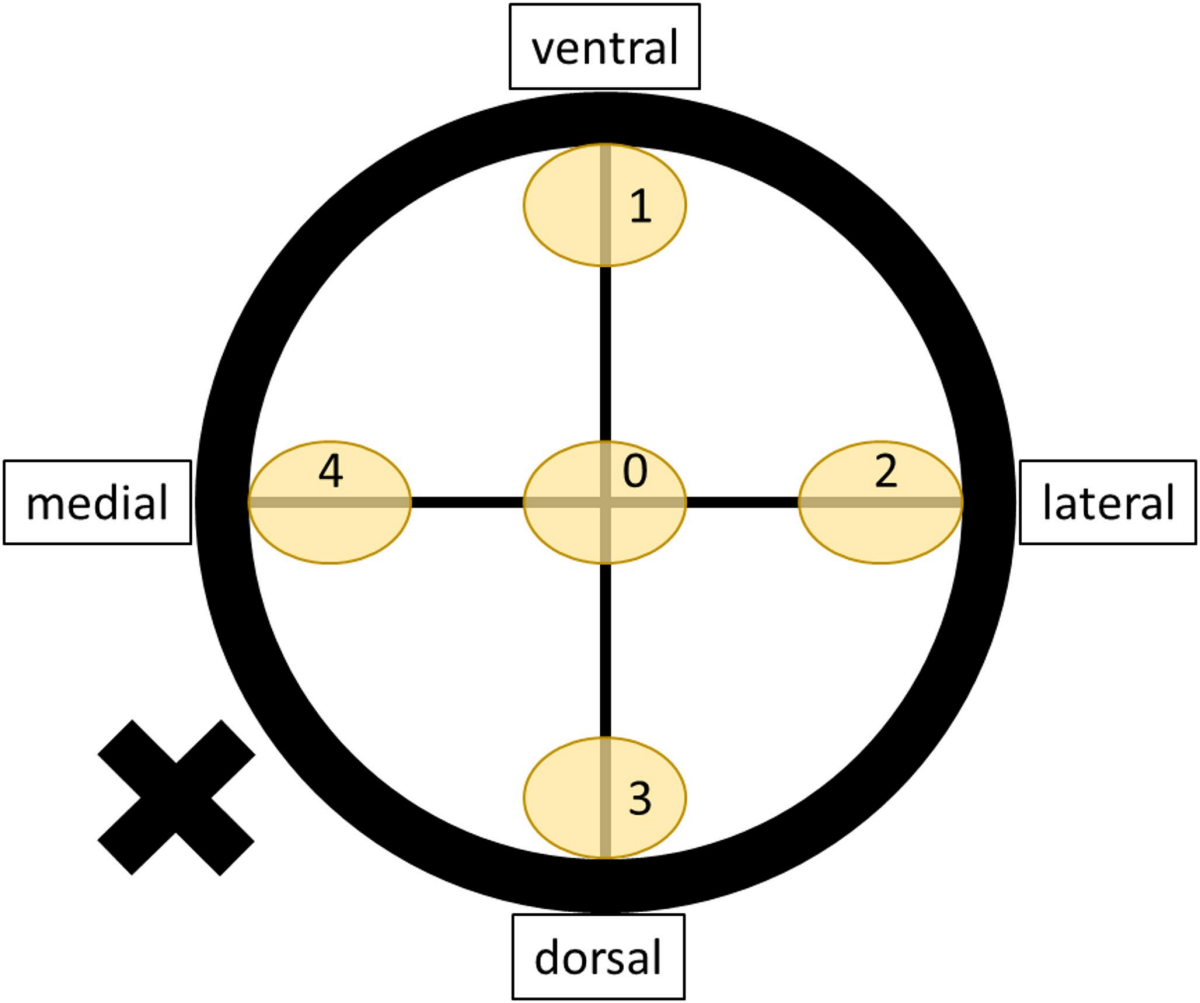
Mounting of the hexapod system to the tibial bone (yellow circle) in five different positions in relation to the reference ring (black circle). The Beacon is mounted between strut 5 and 6 (black cross). Presented is the position of the tibial bone in a bottom-up view

This test setup resulted in five bone positions within the ring system (Fig. 3).

- Position 0 (“neutral”): Center of the bone is in the center of both rings.
- Position 1: Center of the bone is as ventral as possible in the reference ring.
- Position 2: Center of the bone is as lateral as possible in the reference ring.
- Position 3: Center of the bone is as dorsal as possible in the reference ring.
- Position 4: Center of the bone is as medial as possible in the reference ring.

In each position, anteroposterior (ap) and lateral X-rays as well as a CT were performed.

Additionally, to evaluate the influence of the position of the Beacon to the measurements, we also performed X-rays and CTs with the bone in the center position (Position 0) with the Beacon being attached between strut 5 and 6 (Position A) and between strut 3 and 4 (Position B).

### Radiographic test setup

**Fig. 4:**
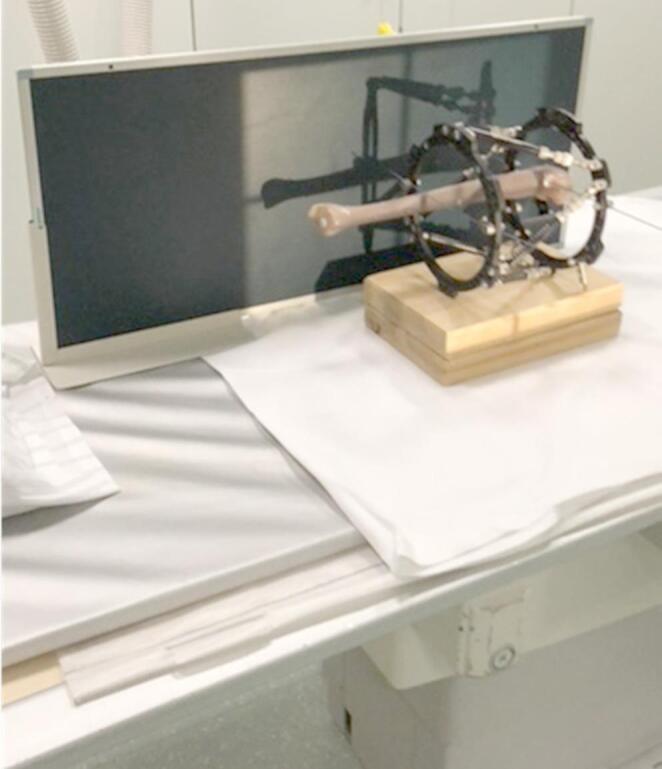
X-ray setup with the hexapod system mounted on the tibial bone. The specimen is positioned at the center of the X-ray plate. The distance between plate and tube was 120 cm

The study was conducted with an artificial bone, therefore, an ethical approval was not applicable. Radiographs were obtained with a 1.3 m-cassette (Global Imaging Baltimore, MD). The X-ray tube-cassette-distance was 120 cm. The specimen was positioned so that the Beacon was in contact with the cassette. The central X-ray beam was aimed at the center between the TSF-rings, both for the anterior-posterior (ap) and the lateral view. The cassette was positioned posterior to the specimen for ap radiographs and medial to the specimen for lateral radiographs (Fig. 4).

**Fig. 5:**
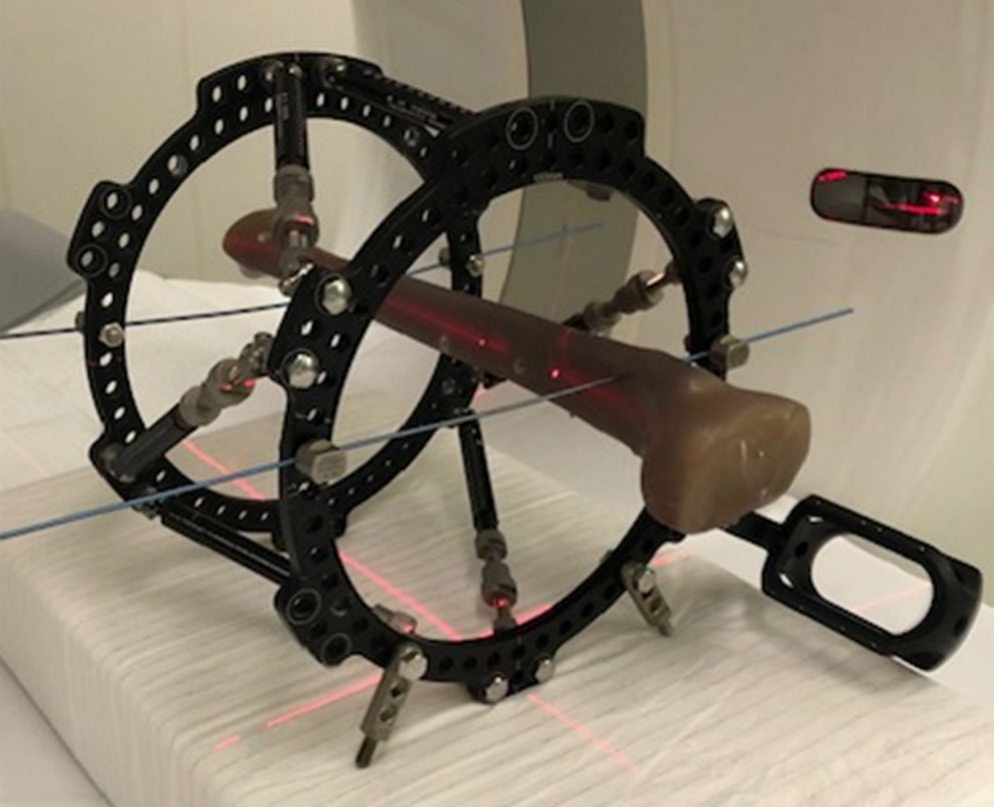
Setup for the CT (computed tomography). The tibial bone mounted in the center of the ring is positioned in the isocenter of the CT

To perform the CTs, the mounted bone was placed in the center of the CT-table and then positioned so that the mounted tibial bone was in the isocenter of the CT (Fig. 5). A SOMATOM Definition Edge (Siemens Healthineers, Munich, Germany) was used.

### Measurements

The CTs were analyzed with AGFA Impax (AGFA HealthCare, Mortsel, Belgium). Total bone length (375 mm) and the eight 50 mm reference distances at the proximal and distal end were measured. Thereafter, the radiographs and distances were analyzed in the SMART-TSF Web Application V6.2. Calibration was done by using the Beacon. All X-rays and CTs were measured by two observers (orthopaedic specialists with at least 6 years of experience in musculoskeletal imaging) independently.

### Statistics

The measured distances of the total bone length and all 50 mm reference distances were noted for each position of the bone (Position 0–4) as well as the two positions A and B of the Beacon with the bone in Position 0. Differences in lengths between the positions of the bone and the Beacon were calculated for X-ray and CT measuring. To answer the secondary aim of our study, descriptive statistics were calculated to describe the measurement variability dependent on the image modality (automatic calibration with the Beacon on X-rays vs. CT). The study data were analyzed with JMP (SAS Institute Inc., 14.2, Cary, NC, USA) and STATA (Stata Corporation, 15.0, College Station, TX, USA).

## Results

The measured total bone length was 375 mm using a sliding caliper. In the standard Position 0, the measured total bone length was 376 mm on the ap and lateral radiograph using the SMART-TSF Web Application. The total bone length measured by CT was 375 mm in all five positions (Position 0–4). Large measurement deviation of the total bone length from 349 mm to 401 mm (-26 mm to + 26 mm; -7 to + 7%) was found for the other bone positions (Position 1–4) when measuring the X-rays with the SMART-TSF Web Application. Resultingly, despite referencing with the Beacon, the measured length varied from the true bone length in ap X-rays between − 17 mm (-5%) and 26 mm (+ 7%) and in lateral X-rays between − 26 mm (-7%) and + 25 mm (+ 7%) (Table 1).

**Table 1:** Measured total bone length in the computed tomography (CT) as well as on the anterior-posterior (ap) and lateral X-ray using the SMART-TSF web Application. Values represent absolute measurement distance and relative to the true bone length of 375 mm

Bone Position	CT		ap X-ray		lateral X-ray	
	Absolute [mm]	Relative [mm]	Absolute [mm]	Relative [mm]	Absolute [mm]	Relative [mm]
0	375	0	376	1	376	1
1	375	0	401	26	371	-4
2	375	0	373	-2	400	25
3	375	0	358	-17	366	-9
4	375	0	376	1	349	-26

**Fig. 6:**
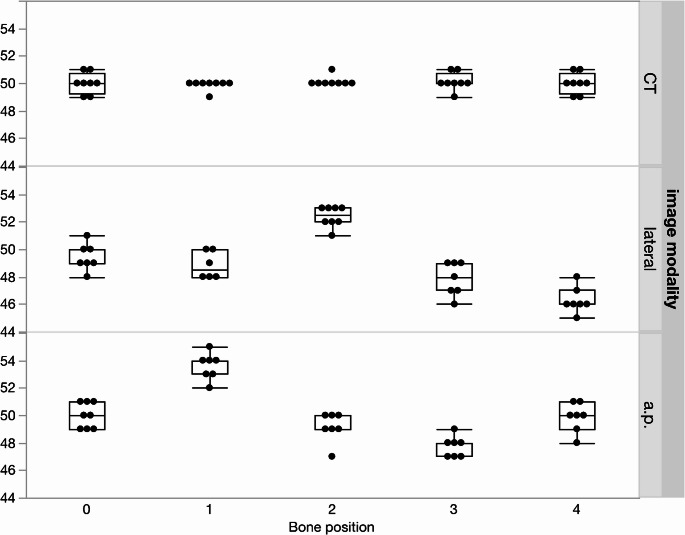
Measured distances and standard deviation for the 50 mm reference distances for all five bone positions (Position 0: center of the ring, Position 1: ventral, Position 2: lateral, Position 3: dorsal, Position 4: medial) in computed tomography (CT), lateral and a.p. X-rays

The data showed that the measurements were dependent on the distance between the center of the reference ring and the bone. Having the bone nearer to the detector in relation to the center of the ring, the measured distance presented shorter and vice versa. Therefore, the bone length was measured shorter in Position 2 and 4 and longer in Position 1 and 3 (Fig. 6).

To evaluate the relevance of the measurement error even at shorter distances, we measured 50 mm reference distances at defined locations on the bone (both at the level of the proximal and distal ring, ventral, dorsal, medial and lateral on the bone). Length measuring of the 50 mm reference distances showed deviations between 47 mm and 55 mm. Thus, a relative measurement error of up to 10% could be found.

In contrast, the reference distances measured by CT showed much less variability (± 1 mm) compared to measurements using radiographs and the SMART-TSF Web Application (Table 2).

**Table 2:** Measured values of the 50 mm reference distances on anterio-posterior X-rays

		Position				
		0	1	2	3	4
Distance [mm]	pv	51	54	50	48	51
	pl	50	53	Not visible	47	49
	pd	49	Not visible	47	Not visible	48
	pm	49	52	49	47	Not visible
	dv	51	55	50	49	51
	dl	51	54	50	48	50
	dd	49	53	49	47	50
	dm	50	54	49	48	50

Positions 0–4 describe the position of the bone within the reference ring (0: standard, 1: ventral, 2: lateral, 3: dorsal, 4: medial). Eight reference distances were marked on the bone and named after their position with two letters. The first letter represents the craniocaudal level. The distances on the level of the proximal ring were named with “p” and the distances on the level of the distal ring were named with “d”. The position on the bone is represented by the second letter “v” (ventral), “d” (dorsal), “l” (lateral) or “m” (medial)

When changing the position of the Beacon between Position A (between strut 5 and 6) to Position B (between strut 3 and 4) with the bone mounted in Position 0, the measured total bone length showed only small variance (373 vs. 376 mm). Furthermore, the measurements of the 50 mm reference distances varied between 48 mm and 51 mm.

## Discussion

Modern hexapod systems with planning softwares are the most accurate and flexible technique to correct complex deformities. However, several requirements are crucial to reach the best accuracy of a deformity correction with the hexapod system [6; 7]. Firstly, the technique of generating the X-rays for deformity correction influences the accuracy of the measurements. Secondly, the obtained X-rays for measuring the mounting parameters influence the results of the planned deformity correction [1; 2]. Therefore, the accuracy of the correction planning and the associated success of the correction is among other things based on the radiologic image acquisition.

The first finding of this study confirms the excellent accuracy of the TSF hexapod system and the associated SMART-TSF Web Application in deformity analyzing if the measured distance is close and parallel to the perpendicular axis of the center of the reference ring. The maximal error between the real and the measured distance on X-rays was 1 mm (2%). This was already confirmed by Ferreira et al. [7]. The present study adds further knowledge that the bone position relatively to the center of the reference ring influences the measurements of distances on X-rays. The change of bone position within the ring from the center to the four extreme positions (ventral, lateral, dorsal and medial) led to clinically relevant measurement deviations of the 50 mm reference distances by up to 5 mm (10%). The longer the distance to be measured, the greater the absolute measurement error if the distance is not in the center of the reference ring. The measured differences in the length measurement of the bone were up to 26 mm from the actual length of 375 mm, depending on the position. If the bone is closer to the detector than the center of the ring, the measured distance is shorter and if the bone is closer to the X-ray source the measured distance is longer. The influence of the magnification device and its position to the resulting measurements on a two-dimensional X-ray film was previously described [2]. By using the Beacon in X-rays, the calibration is made by detecting the Beacon and its dimensions. The software can reference the X-rays with the Beacon and is matching the reference rings´ coordinate system to it very accurately. There is an excellent match of the digital calculated virtual ring to the one shown on the X-ray. Our results are showing, that the calibration is only accurate for the coronal or the sagittal plane on the axis of the center of the reference ring in ap or lateral radiographs, respectively. It is not only in the web application of Smith + Nephew, this calibration is made in every web-based application of hexapod system producers. The problem of measuring inaccuracies is more relevant in cases in which the bone is moving away from the center of the ring. These findings might not be of high relevance in correcting deformities at the femur or the forearm because in these cases the bone is most commonly close to the center of the reference ring. However, using hexapod systems for deformity corrections at the tibia or the humerus, the bone is usually located eccentric. The reason for this is the limb anatomy. If the tibial bone is in the center of the ring system, there would be a big part of the fixator system right between the legs and thus disturbing the ability to walk. If the humeral bone is centered in the ring, a large portion of the ring will be close to the chest or abdomen. Furthermore, with the bone not in the center, the fixation is more stable and rigid because shorter pins can be used. Therefore, the tibia is usually placed in the medial and ventral part of the reference ring and not in the center. Another example of possible measurement errors are extremities with significant axial deviations. In the case of varus or valgus deviations of e.g. 20–30°, parts of the bone will always be outside the center of the reference ring, even if one part of it is mounted directly in the center. Taking the results of this study into consideration, the measurements in these cases might be inaccurate leading to potential inferior deformity correction. An algorithm for correcting measurements for positions outside the reference ring center does not currently exist. Our clinical solution to this problem is the use of CT imaging to plan deformity correction.

We further evaluated the influence of the position of the Beacon on the measured results. Therefore, we changed the position of the Beacon from the standard position between strut 5 and 6 to the position between strut 3 and 4. Only small measurement deviations of 2 mm to the actual bone lengths of 375 mm were measured. Again, these results show that the Beacon is not only a calibration device. It is further used to calculate a coordinate system which is referenced to the center of the reference ring. Since the position of the Beacon is programmed in the software, the center of the reference ring does not change and the measurement results are not affected. The small measurement deviations rather arise from not exactly having mounted the bone in the exact center of the reference ring.

Furthermore, we compared the accuracy of distance measuring between X-rays and CTs. We were able to show excellent accuracy for measuring in CTs for all positions of the bone. As described, for X-rays this is only the case having the measured distance in the center of the reference ring. However, having the bone eccentric, the accuracy is limited. Another problem in X-rays was that some markers were not visible on the radiograph because they were covered by the hexapod hardware which made measuring the distance impossible. By using CTs, despite some radiological artefacts, all distances were exactly measured. Additionally, a clinical problem is arising when using a more rigid double ring system for the reference segment. In these cases, the Beacon is visible in one radiographic plane but covered in the other, so the use of the SMART-TSF Web Application using X-rays is not possible. In these cases, traditional measurements using a reference ball are necessary. Therefore, CTs are superior for detailed planning and resulting in the most accurate distance measurements. In our opinion, X-ray measurements have their importance for therapy monitoring rather than for detailed planning. A disadvantage of CTs is the supine position during acquisition. Long-leg radiographs are performed with full-weight bearing which also influences measurement results [8–10]. What has to be seen critically is the radiation and the dose which is absorbed. To the best of our knowledge, literature for comparing the radiation of X-rays to CTs in deformity correction planning is missing. In our department we are using low-dose CT protocols, which are qualitatively sufficient for planning purposes. A study comparing normal-dose and low-dose CT regarding diagnostic accuracy and radiation dose is already in progress, however, we are not able to provide the results yet.

The study has several limitations. It should be emphasized that this is a radiological study. Its clinical impact of the detected measurements can be debated. As mentioned above, especially for eccentric mounted bones when the measured distance is not located in the center of the reference ring it might lead to wrong deformity correction. Another limitation is that the study used only one bone which was anatomically formed. A deformed bone further influences the distance to the center of the ring which makes estimating the measurement error difficult and unpredictable. Since we only used one bone we are not able to provide statistical testing in order to prove significance. Further research with multiple and deformed bones is needed to analyze this relation. Furthermore, the test setup of the present study was not performed during weight-bearing conditions.

## Conclusion

Care must be taken when using planning softwares for hexapod systems like the SMART-TSF Web Application of Smith + Nephew in cases where the bone is not centered in the reference ring. This applies to tibial and humeral correction as well as severe axis deviations. Large measurement differences can occur. The measurement errors are dependent on the location of the measured distance according to the reference ring und the detector. Having the measured distance nearer to the detector than the center of the reference ring, the distance was measured incorrectly low and vice versa. CT, not susceptible to these typical X-ray projection artifacts, is able to provide a more consistent and accurate measurement regardless of bone position and the position of the reference distances. These findings should be considered in deformity corrections when the limb cannot be centered in the reference ring of the hexapod device.

## Data Availability

All data supporting the findings of this study are available within the paper.
